# Up-regulation of Interferon-inducible protein 16 contributes to psoriasis by modulating chemokine production in keratinocytes

**DOI:** 10.1038/srep25381

**Published:** 2016-05-03

**Authors:** Tianyu Cao, Shuai Shao, Bing Li, Liang Jin, Jie Lei, Hongjiang Qiao, Gang Wang

**Affiliations:** 1 Department of Dermatology, Xijing Hospital, Fourth Military Medical University, Xi’an, China

## Abstract

Psoriasis is a common chronic inflammatory skin disease characterized by epidermal hyperplasia and dermal inflammation. Keratinocyte activation is known to play a critical role in psoriasis, but the underlying mechanism remains unclear. Interferon-inducible protein 16 (IFI16), an innate immune system sensor, is reported to affect keratinocyte function. We therefore hypothesized that IFI16 promotes psoriasis by modulating keratinocyte activation. In the present study, we cinfirmed that IFI16 was overexpressed in epidermal keratinocytes of psoriasis patients. In addition, psoriasis-related cytokines, including IFN-γ, TNF-α, IL-17 and IL-22, induced IFI16 up-regulation in keratinocytes via activation of STAT3 signaling. We also observed that IFI16 activated the TBK1-NF-κB signaling, leading to the production of CXCL10 and CCL20. Importantly, knocking down p204, which is reported as the mouse orthologous of human IFI16, inhibited epidermal hyperplasia in mice with imiquimod-induced psoriasiform dermatitis. These findings indicate that IFI16 plays a critical role in the pathogenesis of psoriasis and may be a potential therapeutic target.

Psoriasis is a common immune-mediated chronic disease affecting skin, joints, or both in 2%–3% of the population worldwide^1^,^2^. It is characterized by redness, thickness and scaling, which seriously undermines the life quality of patients^3^. Although the pathogenesis remains unclear, there is ample evidence to suggest that keratinocyte plays a critical role in this disease^4^.

It is generally accepted that T helper cells, include Th1, Th17 and Th22 cells, contribute to the development of psoriasis by secreting various cytokines including interferon γ (IFN-γ), tumor necrosis factor α (TNF-α), interleukin 17 (IL-17), and IL-22^3^,^4^, which results in the excessive proliferation and aberrant differentiation of keratinocytes. However, recent studies have revealed that keratinocyte is also an important inflammation accelerator in psoriasis. Keratinocyte could sense the damage-associated molecular patterns (DAMPs) or pathogen associated molecular patterns (PAMPs) to be activated. Then, the activated keratinocyte produces various kinds of immune-related proteins such as IL-1α, IL-1β, IL-8, CXCL1, CXCL10, CCL20, ICAM-1, and HLA-DR, leading to an amplified immune response^3^,^4^,^5^,^6^. Thus, keratinocyte activation directly or indirectly causes the major histological features of psoriasis, including epidermal hyperplasia, parakeratosis and skin inflammation.

Interferon-inducible protein (IFI)16, as a member of the IFN-inducible PYHIN-200 gene family in humans, has three isoforms, and p204 has been identified as the mouse orthologous of human IFI16^7^,^8^. IFI16 is expressed in many cell types, including myeloid cells, interstitial cells, endothelial cells and epithelial cells^9^,^10^,^11^,^12^,^13^, and modulates a variety of cell functions, including proliferation, differentiation, apoptosis, senescence and inflammation^7^,^14^. It can not only bind to double-stranded (ds) DNA and single-stranded DNA, but also interact with a variety of proteins such as p53, breast cancer gene 1, retinoblastoma, apoptosis-associated speck-like protein (ASC) and stimulator of interferon genes (STING)^9^,^10^,^15^,^16^. Notably, IFI16 has the ability to initiate the immune response by sensing cytosolic DNA^8^, which leads to the recruitment of STING and the production of type I IFN^17^, or else forms the inflammasome with ASC to induce the production of IL-1β and IL-18^10^. In addition, IFI16 activation induces the production of other cytokines and chemokines, such as IL-6, CXCL10 and CCL20^11^,^18^.

IFI16 has been implicated in the progression of various inflammatory diseases, including systemic lupus erythematosus (SLE), Sjögren’s syndrome (SS) and systemic sclerosis (SSc)^19^,^20^,^21^. Recent studies have shown that IFI16 was also expressed in the cytoplasm and nucleus of primary keratinocytes and overexpressed in the epidermis of psoriasis patients^11^,^22^. However, whether IFI16 plays a pathogenic role in psoriasis and the underlying mechanism remains unknown.

Given that IFI16 is overexpressed in psoriatic keratinocytes and keratinocyte activation is related to psoriasis, we conjectured that IFI16 contributes to the development of psoriasis by modulating keratinocyte function. To test this hypothesis, we assessed IFI16 expression in the lesions of psoriasis patients, and examined the mechanism of IFI16-mediated keratinocyte activation *in vitro*. Further, we used siRNA to knock down p204 in a mouse model of imiquimod (IMQ)-induced psoriasis-like dermatitis, identifying that IFI16 could promote the development of psoriasis *in vivo.*

## Results

### IFI16 is overexpressed in the epidermis of human psoriatic skin

**Figure 1:**
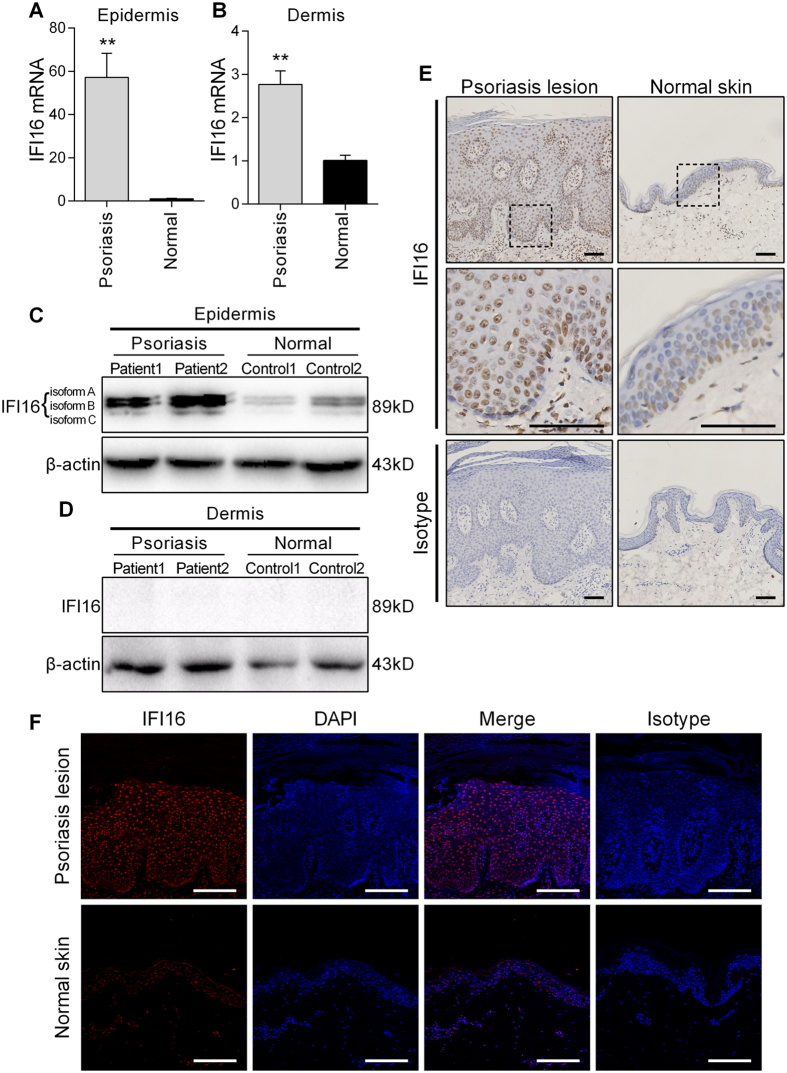
IFI16 expression is increased in psoriasis lesions. (**A**,**B**) mRNA level of IFI16 was evaluated using qPCR in separated epidermis (**A**) and dermis (**B**) of the skin biopsies from 10 psoriasis patients and 10 healthy donors. (**C**,**D**) Protein level of IFI16 were analyzed using western blotting in separated epidermis (**C**) and dermis (**D**) of the skin samples from psoriasis patients and healthy donors. (**E**) Immunohistochemistry staining of IFI16 in normal skin and lesions of psoriasis patients. Bar, 100 μm. (**F**) Immunofluorescence staining of IFI16 (red) in normal skin and psoriatic lesions. Nuclei were counterstained with DAPI (blue). Bar, 100 μm. Values represent mean ± SD. **P < 0.01, vs. normal.

Frist, we examined the expression of IFI16 in the epidermis and dermis of skin samples obtained from psoriasis patients and normal donors. The quantitative (q)PCR results showed that IFI16 mRNA level was significantly increased in both the epidermis and dermis of psoriatic lesions as compared to normal skin samples (Fig.1A,B), while the threshold cycle (CT) value of IFI16 in the dermis of both patients and normal control individuals were extremely high (data not shown), indicating that IFI16 is seldom expressed in the dermis. The western blotting showed that IFI16 protein (three isoforms) was overexpressed in the epidermis of psoriatic lesions (Fig. 1C), but was barely detectable in the dermis (Fig. 1D). Furthermore, the immunohistochemistry and immunofluorescence staining revealed that IFI16 was expressed only in the basal layer of normal skin, but highly expressed in the thickened epidermis of psoriasis patients, primarily in the nuclei of keratinocytes (Fig. 1E,F). These results suggested that IFI16 is overexpressed in the psoriatic lesions, especially in the epidermis.

### Cytokines induce the up-regulation of IFI16 expression in keratinocytes via activation of STAT3 signaling

We next examined the mechanism underlying the up-regulation of IFI16 in keratinocytes of psoriatic epidermis. IFN-γ, TNF-α, IL17, and IL22 are known to induce keratinocyte activation in psoriasis^3^,^4^; the presence of dsDNA is also an trigger of psoriasis^23^. We therefore investigated the effect of these factors on IFI16 expression in keratinocytes. As shown in Supplementary Fig. S1, IFN-γ, TNF-α, IL-17 and IL-22 could up-regulate IFI16 expression in human primary keratinocytes in a dose-dependent manner (Supplementary Fig. S1A–D). We then incubated cells with 10 ng/ml IFN-γ, 50 ng/ml TNF-α, 20 ng/ml IL-17A and 20 ng/ml IL-22, and evaluated IFI16 expression at 0 h, 6 h, 12 h, 24 h, 36 h and 48 h. As a result, the mRNA level of IFI16 was up-regulated in the presence of these cytokines in a time-dependent manner and reached to the peak at 24 h (Supplementary Fig. S1E–H).

**Figure 2:**
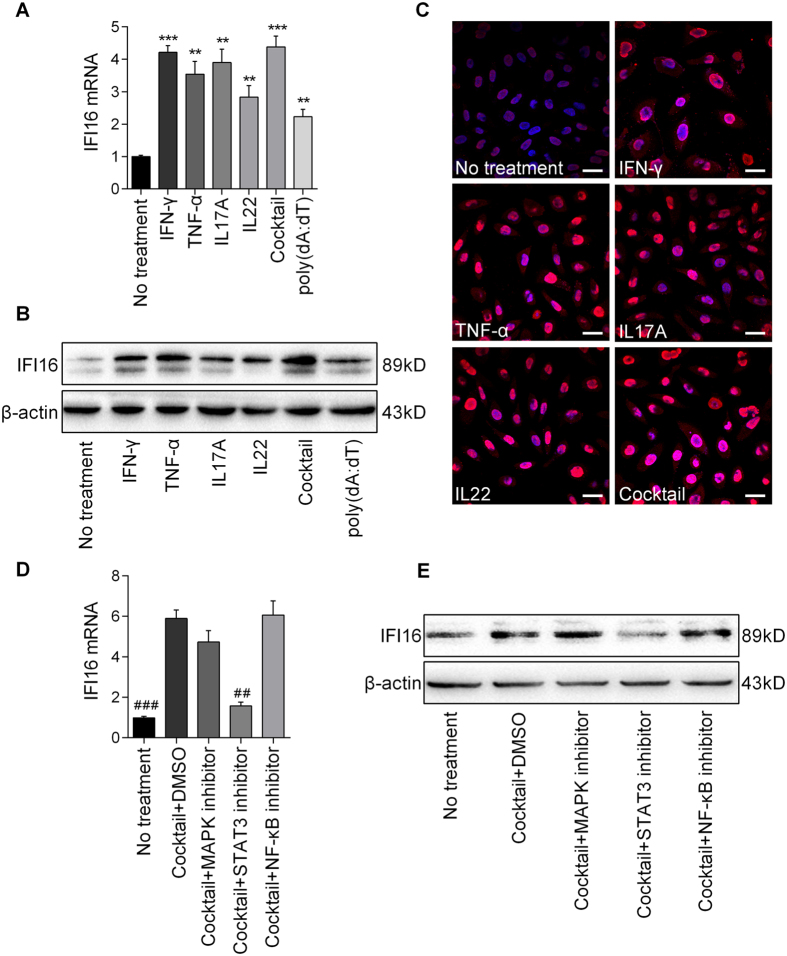
Cytokines up-regulate IFI16 expression in human keratinocytes. (**A**) mRNA and (**B**) protein levels of IFI16 in human primary keratinocytes treated with IFN-γ (10 ng/ml), TNF-α (50 ng/ml), IL-17 (20 ng/ml), IL-22 (20 ng/ml), poly(dA:dT) (2 μg/ml) and a cocktail of the four cytokines for 24 h. (**C**) Immunofluorescence anlaysis of IFI16 expression (red) in human primary keratinocytes treated with IFN-γ, TNF-α, IL-17, IL-22 and the cocktail. Nuclei were counterstained with DAPI (blue). Bar 10 μm. (**D**) mRNA levels and (**E**) protein levels of IFI16 in human primary keratinocytes treated by inhibitors targeting MAPK, STAT3 or NF-κB signaling pathways for 1 h, followed by the cytokine cocktail for 24 h. Values represent mean ± SD. **P < 0.01, ***P < 0.001, vs. no treatment; ^#^P < 0.001, vs. dimethylsulfoxide (DMSO).

Then, the IFI16 expression in human primary keratinocytes cultured with the presence of IFN-γ (10 ng/ml), TNF-α (50 ng/ml), IL17A (20 ng/ml), IL22 (20 ng/ml), poly (dA:dT) (2 μg/ml) or a combination of these cytokines for 24 h was evaluated by qPCR, western blotting, and immunofluorescence. These results showed that IFI16 expression in keratinocytes was up-regulated when treated with each of these components, and most highly expressed with the cytokine cocktail. Besides, cytokines induced IFI16 expression to a greater degree than poly (dA:dT) (Fig. 2A,B). The immunofluorescence analysis revealed that IFI16 was slightly detected in the nuclei of untreated human primary keratinocytes, but obviously overexpressed and localized in both the nucleus and cytoplasm after cytokine stimulation (Fig. 2C).

To identify the signaling pathway by which cytokines modulate IFI16 expression, we pretreated cells with inhibitors targeting mitogen-associated protein kinase (MAPK), STAT3, or NF-κB, and then assessed mRNA and protein levels of IFI16. These results showed that only STAT3 inhibition suppressed cytokine-induced IFI16 expression (Fig. 2D,E), suggesting that psoriasis-related cytokines stimulate IFI16 expression in keratinocytes via the activation of STAT3 signaling.

### IFI16 induces CXCL10 and CCL20 expression *in vitro*

We further investigated the effect of IFI16 on keratinocyte activation by gain- and loss-of-function experiments. Human primary keratinocytes were transfected with a plasmid overexpressing IFI16 or a small interfering (si)RNA construct targeting IFI16 (Supplementary Fig. S2D). Cell proliferation was evaluated by Cell Counting Kit-8 (CCK8), 3-(4,5-dimethylthiazol-2-yl)-2,5-diphenyltetrazolium bromide assay (MTT assay), and 5-ethynyl-2′-deoxyuridine labeling (Edu assay). The results showed that IFI16 overexpression did not induce the proliferation of human primary keratinocytes (Supplementary Fig. S3).

**Figure 3:**
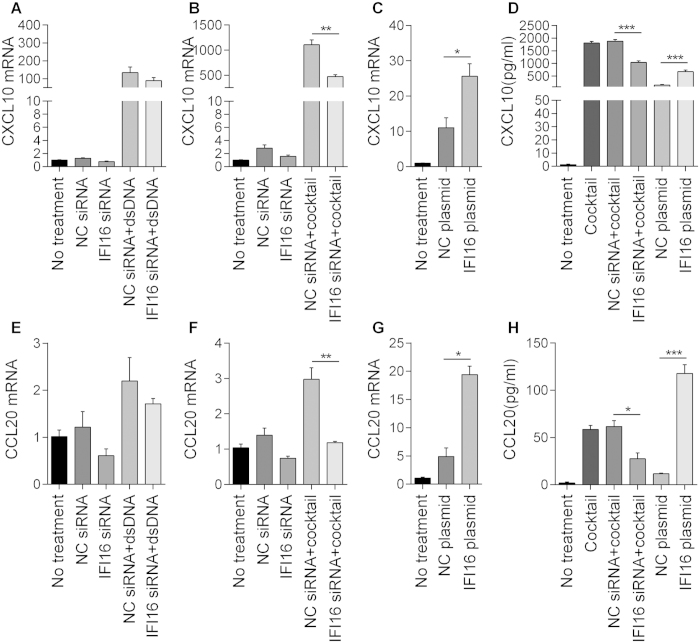
IFI16 induces CXCL10 and CCL20 expression in keratinocytes. Human primary keratinocytes with IFI16-knockin or IFI16-knockdown were treated with poly(dA:dT) (2 μg/ml) or treated with cytokine cocktail for 24 h. mRNA levels of (**A–C**) CXCL10 and (**E–G**) CCL20. Protein levels of (**D**) CXCL10 and (**H**) CCL20.

Previous studies have reported that the expressions of IL-1β, IL-6, IL-18, IFN-α, IFN-β, CXCL10, and CCL20 can be induced by IFI16^8^,^9^,^10^,^11^,^18^. We therefore treated human primary keratinocytes with or without IFI16 knockdown with cytokine cocktail. As the qPCR showed, incubating cells with the cytokine cocktail caused the up-regulation in mRNA levels of IL-1β, IL-6, IL-18, IFN-α, IFN-β, CXCL10, CXCL16, and CCL20; however, knockdown of IFI16 suppressed the expressions of CXCL10 and CCL20 only (Supplementary Fig. S4, Fig. 3B,F).

To confirm the effect of IFI16 on CXCL10 and CCL20 expression in keratinocytes, we treated IFI16-deficient cells with poly(dA:dT) or the cytokine cocktail. Unexpectedly, the qPCR results showed that IFI16-knockdown could not suppress the mRNA expressions of CXCL10 and CCL20 induced by dsDNA (P = 0.272, 0.399, respectively; Fig. 3A,E), but significantly decrease the cytokine cocktail-induced expressions of CXCL10 and CCL20 at both the mRNA and protein levels (Fig. 3B,D,F,H). Besides, the mRNA and protein levels of CXCL10 and CCL20 were also increased in cells overexpressing IFI16 (Fig. 3C,D,G,H). Taken together, these data suggest that IFI16 mediates the high expression of CXCL10 and CCL20 in keratinocytes.

### IFI16 induces the expression of CXCL10 and CCL 20 via the TANK-binding kinase (TBK)1-NF-κB signaling pathway in keratinocytes

**Figure 4:**
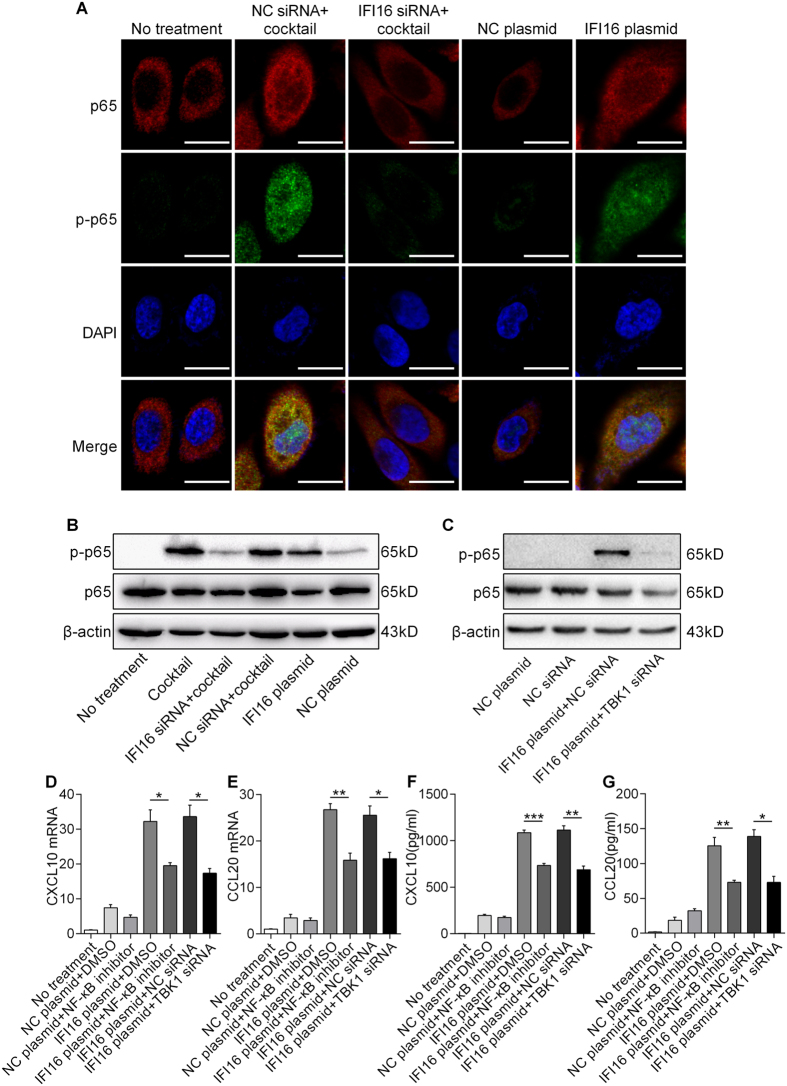
IFI16 promotes CXCL10 and CCL20 expression via TBK1-NF-κB signaling pathway in keratinocytes. (**A**) Immunofluorescence staining of NF-κB p65 (red) and phosphorylation of NF-κB p65 (p-p65) (Green) of human primary keratinocytes treated with IFI16 siRNA and cytokine cocktail, or transfected with IFI16 plasmid. Nuclei were counterstained with DAPI (blue). Bar, 20 μm. (**B**,**C**) The protein levels of NF-κB p65 and the phosphorylation of NF-κB p65 (p-p65). (**D**,**E**) mRNA levels of CXCL10 (**D**) and CCL20 (**E**). (**F**,**G**) Protein levels of CXCL10 (**F**) and CCL20 (**G**). Values represent mean ± SD. *P < 0.05, **P < 0.01, ***P < 0.001.

Given that IFI16 protein up-regulated the expressions of CXCL10 and CCL20 *in vitro*, we determined to investigate the pathway through which IFI16 exerted this effect. It has been proved that CXCL10 and CCL20 could be induced by the activation of the NF-κB signaling^24^,^25^. We therefore investigated whether IFI16 induces the production of CXCL10 and CCL20 via the NF-κB signaling pathway. The expression level of phospho-p65 was increased in human primary keratinocytes overexpressing IFI16 and decreased in those deficient in IFI16, as determined by immunofluorescence and western blotting (Fig. 4A,B). Moreover, pre-treating IFI16-overexpressing cells with NF-κB inhibitor reduced both mRNA and protein levels of CXCL10 and CCL20 (Fig. 4D–G). These results suggest that IFI16 induces CXCL10 and CCL20 expressions via activation of NF-κB signaling in keratinocytes.

On the other hand, the TBK1 has been well explored as the downstream molecule of IFI16^11^. Thus, we also explored whether IFI16 activated the NF-κB signaling through TBK1. We treated IFI16-overexpressed keratinocytes with the siRNA targeting TBK1, and found that the phosphorylation of p65 was restrained (Fig. 4C) and secretion of CXCL10 and CCL20 were reduced (Fig. 4D–G). These results indicate that IFI16 induces CXCL10 and CCL20 expressions via TBK1-NF-κB signaling in keratinocytes.

### Local depletion of p204 alleviates psoriatic lesions

**Figure 5:**
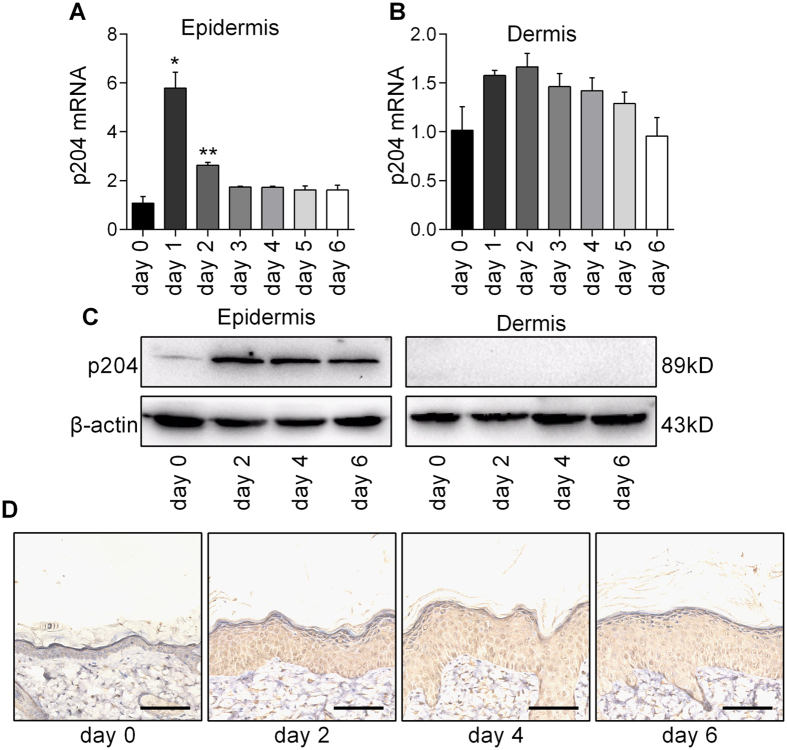
P204 is overexpressed in the IMQ-induced psoriasis-like mice. Local application of IMQ was performed on the back of mice daily for 6 continuative days. Skin samples were collected at day 0, 1, 2, 3, 4, 5 and 6. (**A**,**B**) mRNA level of p204 in epidermis (**A**) and dermis (**B**). (**C**) Protein level of p204 was analyzed by western blotting. (**D**) Immunohistochemistry staining of p204. Values represent mean  ± SD. *P < 0.05, **P < 0.01, vs. day 0.

To determine whether IFI16 plays a critical role in the pathogenesis of psoriasis, we explored the function of p204—the mouse orthologous of the human IFI16 protein as reported in several studies^7^ ‒in a mouse model of IMQ-induced psoriasis-like dermatitis. We firstly examined the expression of p204 in the epidermis and dermis in IMQ-induced mice. The p204 transcript was up-regulated in the epidermis after IMQ treatment, reaching a peak on day 1 (Fig. 5A), but was unaltered in the dermis (Fig. 5B). Similar results were observed for p204 protein between day 2 and 6 (Fig. 5C). The immunohistochemistry analysis also revealed that p204 expression was increased in the epidermis of mice’s skin lesions after IMQ treatment (Fig. 5D), implying that IFI16 (p204) contributed to the development of psoriasis not only at a very early stage, but also throughout the whole disease progression.

**Figure 6:**
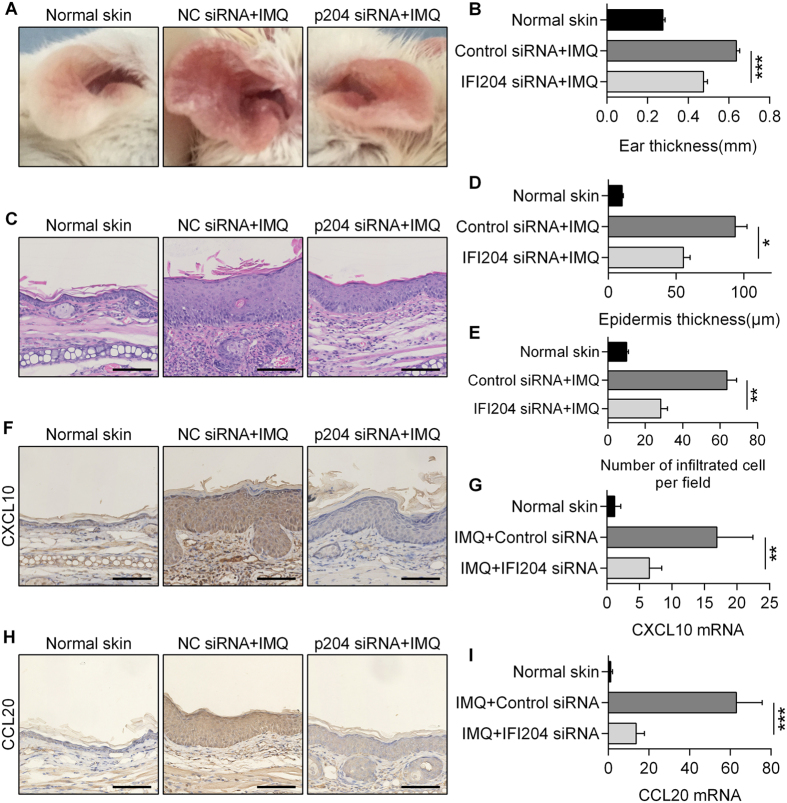
Blocking p204 expression ameliorates epidermal hyperplasia in IMQ-treated mice. The ears of mice were treated with p204 siRNA or NC siRNA every 48 h, and with IMQ every 24 h. (**A**) Psoriatic syndrome. (**B**) Ear thickness. (**C**) HE staining. Bar, 100 μm. (**D**) Epidermal thickness. (**E**) Number of infiltrated lymphocytes in the dermis (400× magnification). (**F**,**H**) Immunohistochemical analysis of CXCL10 (**F**) and CCL20 (**H**). Bar, 100 μm. (**G**,**I**) mRNA levels of CXCL10 (**G**) and CCL20 (**I**) in the epidermis. Values represent mean ± SD. *P < 0.05, **P < 0.01, ***P < 0.001.

**Figure 7:**
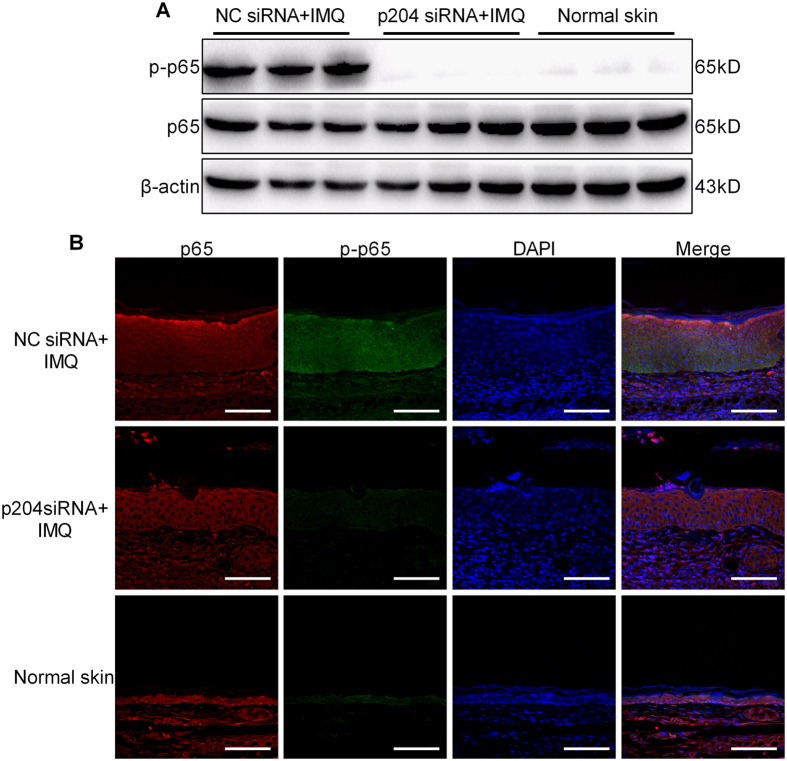
Blocking p204 expression suppresses activation of NF-κB signaling in IMQ-treated mice. (**A**) Protein levels of p65 and phosphor-p65 in skin lesions from mice treated with p204 or NC siRNAs. (**B**) Immunofluorescence staining of p65 (red) and phosphor-p65 (green). Nuclei were counterstained with DAPI (blue). Bar, 100 μm.

Subsequently, p204 siRNA was locally applied every 48 h to the ear skin of IMQ-treated mice to suppress p204 levels in the epidermis, with the interference efficiency determined by western blotting and immunohistochemistry (Supplementary Fig. S5). Notably, p204 knockdown ameliorated psoriatic lesions (Fig. 6A), caused a decrease in ear thickness (Fig. 6B), reversed epidermal hyperplasia (Fig. 6C,D), and reduced the number of inflammatory cells in the dermis (Fig. 6E). Importantly, CXCL10 and CCL20 expressions were decreased in the psoriasis-like lesions in the absence of p204 (Fig. 6F–I), which was accompanied by a decrease in the phospho-p65 level and inhibition of p65 nuclear translocation in epidermal cells (Fig. 7A,B). Taken together, these data receal that local depletion of IFI16 ameliorates psoriatic lesions *in vivo*.

## Discussion

This study demonstrated that IFI16 was highly expressed in the epidermis of lesions from psoriasis patients. *In vitro* results showed that several psoriasis-related cytokines, including IFN-γ, TNF-α, IL-17 and IL-22, could up-regulate IFI16 expression in keratinocytes via activation of STAT3 signaling. Moreover, IFI16 induced CXCL10 and CCL20 production in keratinocytes via TBK1-NF-κB signaling. Importantly, in a mouse model of psoriasis, p204 knockdown improved epidermal hyperplasia, alleviated skin inflammation and reduced the expression levels of CXCL10 and CCL20. These results indicate that IFI16 contributes to the pathogenesis of psoriasis by modulating keratinocyte activation.

Keratinocyte activation is thought to be critical for the initiation and acceleration of psoriasis^3^,^4^. Recent studies have shown that danger-associated molecular patterns (DAMPs) and inflammatory cytokines can activate keratinocytes to produce cytokines and chemokines, respectively^3^,^4^,^11^,^23^. However, the mechanism of keratinocyte activation in psoriasis is not well understood. Chiliveru *et al.* have found that IFI16 is up-regulated in psoriatic skin lesions and localized to the cytoplasm in a subpopulation of cells. In our study, we confirmed that IFI16 was overexpressed in the epidermis of psoriatic patients, and *in vivo* evidences suggested that knocking down p204 in imiquimod-treated mice successfully improved epidermal hyperplasia and alleviated skin inflammation, which demonstrated that IFI16 contributes to psoriasis development and may be targeted in psoriasis treatment.

IFI16 belongs to the IFN-inducible PYHIN-200 gene family and acts as a DNA sensor in many cell types, including dendritic cells, myeloid cells, B cells and keratinocytes^9^,^17^,^26^,^27^. IFI16 overexpression has been observed in epidermal lesions of various autoimmune diseases, such as SLE, SSc, and psoriasis^11^,^22^,^28^,^29^. However, no prior studies have addressed the pathogenetic role of IFI16 in these diseases. We found that besides cytosolic dsDNA, the psoriasis-related cytokines IFN-γ, TNF-α, IL-17 and IL-22 also induced the up-regulation of IFI16 in keratinocytes via STAT3 signaling, which is partly consistent with a recent study demonstrating that TNF-α and IL-1β enhance IFI16 expression induced by cytosolic dsDNA in human primary keratinocytes^11^,^28^,^29^. In addition, our *in vivo* data suggested that p204 expression was up-regulated in epidermal keratinocytes in both the early stage of psoriasis and throughout the disease progression.

IFI16 has been shown to promote type I IFN production through the STING-TBK1-IRF3 signaling pathway^30^. Previous studies have reported that after treatment with cytosolic DNA together with TNF-α or IL-1β, IFI16 colocalized with DNA and STING in the cytoplasm and recruited TBK1 in keratinocytes^11^. In addition, IFI16 also has the ability to recruit ASC and forms a functional IFI16-ASC inflammasome, producing the functional IL-1β and IL-18^10^,^31^. Notably, our *in vitro* and *in vivo* data indicated that IFI16 was not involved in cytokine-mediated production of type I IFNs, IL1β, or IL18 in keratinocytes, but was required for the expression of CXCL10 and CCL20, which are also important factors in the immune response of psoriatic lesions. For instance, CXCL10 is detected in both skin lesions and serum obtained from psoriasis patients, and decreasing the expression of CXCL10 successful improved the psoriatic active plaques^32^,^33^. Moreover, an increase in lesional CCL20 expression is found to incude the recruitment of CCR6^+^ helper T 17 cells in psoriasis^34^. Given that IFI16 can be up-regulated by T cell-derived cytokines as previously described, a “T cells-IFI16-chemokines-T cells” loop may exist in psoriatic lesion, which leads to the enhancement of local inflammation and contributes to the progression of psoriasis.

The NF-κB signaling pathway is reported as the upstream of CXCL10 and CCL20^24^,^25^, and is activated in keratinocytes of psoriatic lesions^35^,^36^. In agreement with the observation that IFI16 can mediate NF-κB activation^30^, we found that IFI16 promotes the phosphorylation of the NF-κB p65 subunit and its translocation into the nucleus both *in vitro* and *in vivo*, and that inhibiting the NF-κB pathway abrogated IFI16-induced up-regulation of CXCL10 and CCL20 expression. Moreover, blocking TBK1 expression in IFI16-overexpressed keratinocytes suppressed the activation of NF-κB signaling and reduced the expression of CXCL10 and CCL20, confirming that IFI16 modulates these important factors through TBK1-NF-κB signaling pathway. Therefore, our results indicate that IFI16 plays a critical role in keratinocyte activation in psoriasis.

Accumulated studies have indicated that the subcellular localization of IFI16 is likely to determine its function^37^, and cytoplasmic IFI16 is tended to evoke immune response by sensing cytosolic DNA^38^,^39^. In psoriatic lesions, cytoplasmic IFI16 is detected in 5–8% of cells, indicating it may reflect immune response in psoriasis^11^. However, exposure of keratinocytes to ultraviolet B light causes the translocation of IFI16 protein from nucleus to cytoplasm, leading to cell apoptosis rather than activation of an immune response^40^. We found that IFI16 was up-regulated in the epidermis of psoriatic lesions and is mainly localized in the nucleus. Therefore, we focused on the biologic functions of IFI16 in the progression of psoriasis, but not its subcellular localization. Reconciling these observations and clarifying the mechanism of immune activation by IFI16 will be the focus of future studies.

In conclusion, we demonstrate that the cytokines-mediated overexpression of IFI16 contributes to the pathogenesis of psoriasis by activating the TBK1-NF-κB signaling pathway and then inducing the production of CXCL10 and CCL20 in keratinocytes. Our findings consider IFI16 functions as a central hub in the initiation of immune response in psoriasis. Nevertheless, further studies of IFI16 are needed to fully clarify the pathogenic role of IFI16 and evaluate its potential to be a therapeutic target in psoriasis.

## Methods

### Patients and skin samples

Psoriasis patients with a dermatologist-confirmed diagnosis of chronic plaque psoriasis (12 females and 9 males; age range, 18–59 years; mean age, 32.2 years) and normal volunteers (6 female and 5 male; age range, 21–43 years; mean age, 31.2 years) were recruited for this study. Patient information is shown in Supplementary Table S1. The psoriasis patients required Psoriasis Area Severity Index (PASI) of at least 8 and a typical lesion of at least 1 cm in size that was suitable for biopsy. One 5 mm biopsy was obtained from each normal volunteer. All of the patients enrolled in our study had no other autoimmune or systemic diseases, and the target lesion and surrounding 5 cm area were not treated with any therapeutic measures for at least 2 weeks before the biopsy. Skin samples were incubated with 2.5 mg/ml dispase (Gibco, Grand Island, NY, USA) for 8 h at 4 °C to separate the epidermis from the dermis. All participants provided written, informed consent for their participation. The study protocol was designed and carried out according to the principles of the Declaration of Helsinki and was approved by the ethics review board of the Fourth Military Medical University.

### Mice

All experimental protocols were performed in accordance with NIH guidelines and were approved by the Review Committee for the Use of Animals of the Fourth Military Medical University. Female BALB/c mice aged 6–8 weeks were purchased from the Department of Laboratory Animal Medicine of the University. Mice were treated daily with Aldara IMQ Cream (iNova, Chatswood, Australia) to induce a psoriasis-like mouse model^41^. To detect time-dependent expression of p204 in mice with the IMQ-induced psoriasis-like dermatitis, IMQ was locally applied to the shaved back of mice. Lesions were collected after 0, 1, 2, 3, 4, 5 and 6 days, respectively. To investigate the effect of p204 blockade on psoriasis, the siRNA targeting p204 (Ribobio, Guangzhou, China) for mice was modified by –OMe and –Chol to increase the stability and lipotropy of p204 siRNA molecule *in vivo.* We used emulsion matrix to blend the siRNA targeting p204 and topically applicated it on the ear skin of mice. For each mouse, the left ear was treated with 2 nmol siRNA mixed with 5 mg emulsion matrix every 48 h. The negative control (NC) siRNA was applied on the right ear in the same way. Both ears were treated with IMQ daily for continuative 6 days. On day 6, skin specimens were obtained from the mice for analysis. The p204 siRNA sequence was as follows: sense, 5′-CCGAAAGAACACAAUCUAUdTdT-3′; antisense, 3′-dTdTGGCUUUCUUGUGUUAGAUA-5′.

### Cell lines and reagents

Human primary keratinocytes were obtained from prepuces obtained from healthy individuals who accepted circumcision. Informed consent was obtained from all donors. Keratinocytes grown to 40–60% confluence were stimulated with IL-17A (20 ng/ml), IL22 (20 ng/ml), IFN-γ (10 ng/ml), TNF-α (50 ng/ml), or a mixture of these cytokines (cocktail) for 48 h and then analyzed. For inhibition experiments, cells were pretreated with inhibitors targeting MAPK (PD98059) (Cell Signaling Technology, Beverly, MA, USA), STAT3 (Santa Cruz Biotechnology, Dallas, TX, USA), and NF-κB (PDTC) (Beyotime, Shanghai, China) for 1 h to block these signaling pathways.

### SiRNAs and plasmids

Human primary keratinocytes were transfected with siRNAs targeting IFI16 and TBK1 (Q000003428, Q000029110; Ribobio, Guangzhou, China) or with plasmid encoding IFI16 (RC202193; OriGene, Rockville, MD, USA) according to the manufacturer’s instructions using Lipofectamine 3000 (Invitrogen, Carlsbad, CA, USA).

### Quantitative PCR (qPCR)

Total RNA was isolated from cells or tissues using a Total RNA Extraction kit (Anmei Biologicals, Xi’an, China) and used to synthesize cDNA with the PrimeScript RT Reagent kit (Takara Bio, Ohtsu, Japan) according to the manufacturers’ protocols. qPCR was performed as described in Supplementary Information online. Expression levels were normalized to that of glyceraldehyde 3-phosphate dehydrogenase (GAPDH). Primer sequences are showed in Supplementary Table S2.

### Western blotting

Cells or tissues were lysed with cell lysis solution (DSL, Webster, TX, USA). The total protein concentration in lysates was measured with the bicinchoninic acid assay (Pierce, Rockford, IL, USA). Equal amounts of protein were separated by sodium dodecyl sulfate polyacrylamide gel electrophoresis and then transferred to nitrocellulose membranes, which were probed with antibodies against IFI16 (Abcam, Cambridge, UK), p204 (Thermo Fisher Scientific, Waltham, MA, USA), p65 (Abcam) and phospho-p65 (Cell Signaling Technology). The information for these antibodies are shown in Supplementary Table S3.

### Immunohistochemistry

Skin tissues from human donors or mice were fixed in 4% formalin buffered solution and embedded in paraffin. 5 mm tissue sections were staining with hematoxylin and eosin (H&E). Immunohistochemistry was carried out as described in Supplementary information online. Sections were labeled with antibodies against p204 (Thermo Fisher Scientific), IFI16, CXCL10, and CCL20 (all from Abcam). The information for these antibodies are shown in Supplementary Table S3.

### Immunofluorescence

Immunofluorescence analysis of skin tissue sections and cultured cells were carried out as described in Supplementary information online. Tissue sections or cells were labeled with antibodies against IFI16, p65, and phospho-p65. The information for these antibodies are shown in Supplementary Table S3.

### Enzyme-linked immunosorbent assay (ELISA)

CXCL10 and CCL20 levels in cell culture media were detected using ELISA kits (Cusabio, Wuhan, China) according to the manufacturer’s instructions.

### Statistical analysis

Each experiment was performed at least three times, and data were analyzed with the unpaired, two-tailed Student’s t-test or by one-way analysis of variance using GraphPad Prism v.6.0 (GraphPad, La Jolla, CA, USA). Data were expressed as the mean ± SD. P-values < 0.05 were considered statistically significant.

## Additional Information

**How to cite this article**: Cao, T. *et al.* Up-regulation of Interferon-inducible protein 16 contributes to psoriasis by modulating chemokine production in keratinocytes. *Sci. Rep.* **6**, 25381; doi: 10.1038/srep25381 (2016).

## Supplementary Material

Supplementary Information
