# Author Correction: Up-regulation of Interferon-inducible protein 16 contributes to psoriasis by modulating chemokine production in keratinocytes

**DOI:** 10.1038/s41598-020-72790-1

**Published:** 2020-09-25

**Authors:** Tianyu Cao, Shuai Shao, Bing Li, Liang Jin, Jie Lei, Hongjiang Qiao, Gang Wang

**Affiliations:** Department of Dermatology, Xijing Hospital, Fourth Military Medical University, Xi’an, China

Correction to: *Scientific Reports* 10.1038/srep25381, published online 03 May 2016

This Article contains an error in Figure 7A, where the image of the western blot for β-actin is a duplication of the image for p65. The correct Figure 7A appears below as Figure 1.

**Figure 1:**
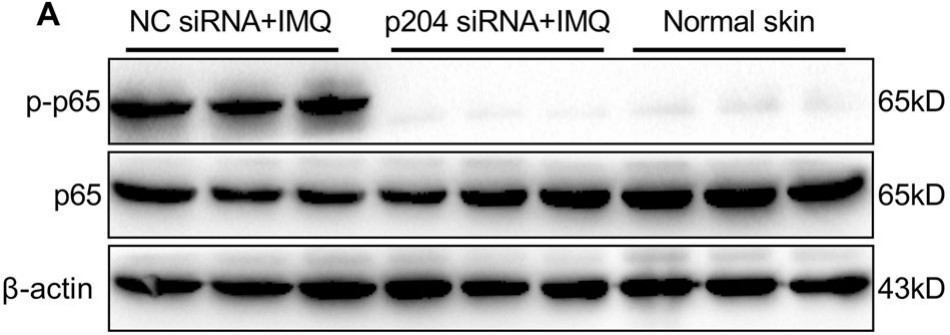
Blocking p204 expression suppresses activation of NF-κB signaling in IMQ-treated mice. (**A**) Protein levels of p65 and phosphor-p65 in skin lesions from mice treated with p204 or NC siRNAs.

