# Supplementary material for: Up-regulation of Interferon-inducible protein 16 contributes to psoriasis by modulating chemokine production in keratinocytes
Source: Sci Rep. 2016 May 3;6:25381. doi: 10.1038/srep25381 (PMC4853747; doi:10.1038/srep25381)

Supplementary information

Up-regulation of Interferon-inducible protein 16 contributes to psoriasis by modulating chemokine production in keratinocytes

Tianyu Cao[1, 2], Shuai Shao[1, 2], Bing Li[1, 2], Liang Jin[1], Jie Lei[1], Hongjiang Qiao[1] and Gang Wang[1, *]

[1] Department of Dermatology, Xijing Hospital, Fourth Military Medical University, Xi'an, China

[2] These authors equally contributed to this paper

*Corresponding author: Department of Dermatology, Xijing Hospital, Fourth Military Medical University, Xi'an, Shaanxi Province, China. Tel. & fax: +86-29-84775401.

E-mail address: xjwgang@fmmu.edu.cn (Gang Wang)

Supplementary materials and methods

Cell lines and reagents Human primary keratinocytes were cultured in the serum-free keratinocyte growth medium (Gibco, NY, USA), and the second- or third-passage keratinocytes were used in all experiments. For observing the does-dependent manner of cytokine-mediated IFI16 expression, cells at 40–60% confluence were stimulated with different concentration of IL-17A, IL22, IFN-γ or TNF-α (0 ng/ml, 10 ng/ml, 20 ng/ml, 30 ng/ml, 40 ng/ml, 50 ng/ml). After 24 h, cells were collected for qPCR detection. As the time dependent manner, cells at 40–60% confluence were stimulated with IL-17A (20 ng/ml), IL22 (20 ng/ml), IFN-γ (10 ng/ml), TNF-α (50 ng/ml) (Peprotech, Rocky Hill, NJ, USA).The cells were collected after 0, 6, 12, 24, 36 and 48 h for qPCR assay, respectively.

siRNAs and Plasmid IFI16 siRNA, TBK1 siRNA and NC siRNA (Q000003428, Q000029110, siN05815122147; Ribobio, Guangzhou, China) were dissolved in diethylpyrocarbonatetreated H_2O at a concentration of 20 mmol/l as a stock. According to the manufacturer's instructions, human primary keratinocytes were transfected with siRNAs (50 nM) using Lipofectamine 3000 (Invitrogen, Carlsbad, CA, USA) 24 h before treatment cytokines.

The plasmid targeting IFI16 and the NC plasmid (pCMV6-Entry) were purchased from OriGene (RC202193, PS100001; Rockville, MD, USA) and were extracted using Plasmid kit (Omega Bio-Tek, Norcross, GA, USA) according to the manufacturer's instructions. Human primary keratinocytes were transfected with these

plasmids for 24 h before treatment with cytokines.

Quantitative PCR (qPCR) Total RNA of cells or tissues was isolated using the Total RNA Extraction Kit (Anmei Biologicals, Xi'an, China) following the manufacturer's instructions, and cDNA was synthesized with PrimeScript RT reagent Kit (Takara, Ohtsu, Japan) according to the manufacturer's protocol. qPCR was performed using IQ SYBR Green Supermix (Bio-Rad, Hercules, CA, USA) on a Chromo continuous fluorescence detector with a PTC-200 DNA Engine Cycler (Bio-Rad). Primer sequences were showed in Supplementary Table S1. The resulting amplification and melt curves were analyzed to ensure the identity of the specific PCR products. Relative quantification was performed according to the ΔΔCT method, and the results were expressed in the linear form using the formula $2^{-\Delta\Delta CT}$. Results were considered significant when at least a 2-fold difference in expression levels was detected.

Western blotting Total proteins of cells or tissues were lysed with cell lysis solution (DSL, Webster, TX, USA). Equal amounts of proteins were separated by 10% SDS-PAGE (Bio-Rad) and were then transferred from the gel to polyvinylidene difluoride membranes (Millipore, Billerica, MA, USA). Page Ruler Plus Prestained Protein Ladder (Fermentas, Hanover, MD, USA) was used to confirm protein electrophoresis and transferring. After blocking in a solution of 5% non-fat dry milk diluted in Tris-buffered saline, the membranes were washed and then incubated with primary antibodies for 12 h at 4 °C. After 3 times washing, the membranes were

incubated with horseradish peroxidase-conjugated secondary antibodies (Goat Anti-Mouse IgG, 1 : 5000; Goat Anti-Rabbit IgG, 1 : 5000; Donkey Anti-Goat IgG, 1 : 5000, Cwbio, Beijing, China) for 2 h at room temperature. The results were detected by the ECL western blotting detection system (Millipore).

Immunohistochemistry The sections of skin tissues were fixed in 4% formalin buffered solution and embedded in paraffin. 4 μm sections of paraffin embedded skins were blocked at room temperature with 5% goat serum in PBS for 30 min, and incubated with primary antibodies at 4 °C overnight, followed by HRP labeled goat anti-mouse/rabbit antibody (Cwbio) for 30 min at room temperature. DAB revelation (Gene tech, Shanghai, China) was used for detecting the biotinylated antibodies.

Immunofluorescence The sections of skin tissues were fixed in 4% formalin buffered solution and embedded in paraffin. Immunofluorescence staining was performed by incubating the paraffin sections with primary antibodies overnight at 4 °C, followed by 1 h incubation with appropriate secondary antibodies (Goat anti-Mouse IgG, Cy3 Conjugated, 1 : 500; Goat anti-Rabbit IgG, FITC Conjugated, 1 : 500; Donkey anti-Goat IgG, Cy3 Conjugated, 1 : 500 and Donkey anti-Rabbit IgG, FITC Conjugated, 1 : 500, Cwbio). DAPI (Dako, Glostrup, Denmark) was used as a counterstain. Tissue sections were analyzed by FV-1000/ES confocal microscope (Olympus America, Melville, NY, USA).

Human primary keratinocytes for immunofluorescence staining were first seeded

on coverslips in a 24-well plate and allowed to adhere before staining. Cells were washed with phosphate-buffered saline (PBS), fixed with 4% paraformaldehyde for 15 min, and permeabilized with 10% saponin for 5 min followed by acetone treatment at −20 °C for 10 min. After blocking with normal goat sera at room temperature for 1 h, cells were incubated with primary antibody diluted in the blocking solution at 4 °C overnight. Cells were washed with PBS and incubated with secondary antibodies at room temperature for 1 h. Nuclei was stained with DAPI (Dako) and subsequently analyzed by FV-1000/ES confocal microscope (Olympus America).

Cell proliferation assay Human primary keratinocytes (1×10^3 per well) were seeded in 96-well culture plates and transfected with IFI16 plasmid or NC plasmid. After 24 h, the proliferation of cells was detected using cell counting kit (CCK)-8 (Beyotime, Shanghai, China) or EdU (5-ethynyl-2'-deoxyuridine) assay kit (Ribobio). 3-(4,5-dimethyl-2-thiazolyl)-2,5-diphenyl-2H-tetrazolium bromide (MTT; Sigma, St. Louis, MO, USA) was performed at 12 h, 24 h, 48 h and 72 h after plasmid transfection following manufacturer's instructions.

Supplementary Tables

Table S1. Information of psoriasis patients

Number	Sex	Age	PASI
1	females	26	10.3
2	females	22	14.6
3	females	51	18.9
4	females	40	9.7
5	females	27	12.9
6	females	26	27.2
7	females	51	24.1
8	females	19	27.5
9	females	18	13.5
10	females	25	18.2
11	females	30	13.2
12	females	31	15.5
13	males	45	9.2
14	males	38	8.9
15	males	22	12.9
16	males	25	16.6
17	males	33	15.2
18	males	29	16.9
19	males	44	19.8
20	males	36	22.1
21	males	38	14.5

Table S2. Sequences of primers

Primer		Sequences	Annealing Temperature
Human IFI16	Sense	5’-TAGGCCCAGCTGAGAGCCATCC-3’	57°C
	Antisense	5’-TGAGGTCACTCTGGGCACTGTCTT-3’	
Human CXCL10	Sense	5’-CCTCCAGTCTCAGCACCAT-3	56°C
	Antisense	5’-AAATTGGCTTGCAGGAATA-3’	
Human CCL20	Sense	5’-TACTCCACCTCTGCGGCGAATCAGAA-3’	56°C
	Antisense	5’-GTGAAACCTCCAACCCCAGCAAGGTT-3’	
Human IFN-α1	Sense	5’-CAGAGTCACCCATCTCAGCA-3’	56°C
	Antisense	5’-CCATCAGACAGGAGGAAGGA-3’	
Human IFN-α2	Sense	5’-ATCTGCTGCTTGGGATGAGA-3’	56°C
	Antisense	5’-TGATTTCTGCTCTGACAACCTC-3’	
Human IFN-β	Sense	5’-TCTCCTGTTGTGCTTCTCCA-3’	57°C
	Antisense	5’-TCTCATTCCAGCCAGTGCTA-3’	
Human IL-1β	Sense	5’-CTGTCCTGCGTGTTGAAAGA-3’	57°C
	Antisense	5’-TTCTGCTTGAGAGGTGCTGA-3’	
Human IL-6	Sense	5’-CTTCGGTCCAGTTGCCTTCT-3’	57°C
	Antisense	5’-AGTGCCTCTTTGCTGCTTTC-3’	
Human IL-18	Sense	5’-GAACCAGTAGAAGACAATTGC-3’	56°C
	Antisense	5’-GGTCTCTCTCTTTTTCACAAGC-3’	
Human CXCL1	Sense	5’-GCCCAAACCGAAGTCATAGCC-3’	56°C
	Antisense	5’-ATCCGCCAGCCTCTATCACA-3’	
Human CXCL16	Sense	5’-GGCCCCTCATTTAAAAACGG-3’	57°C
	Antisense	5’-GCCTGGTCAACATGGTGAAAC-3’	

Hunan GAPDH	Sense	5’-TCTTTTGCGTCGCCAGCCGAG-3’	57°C
	Antisense	5’-ACCAGGCGCCCAATACGACCA-3’	
Mouse p204	Sense	5’-AAAGAGCAAGTTCATCCCAAGA-3’	56°C
	Antisense	5’-GCCTGGTTCACACCTGACAT-3’	
Mouse CXCL10	Sense	5’-TTTCTGCCTCATCCTGCTG-3’	57°C
	Antisense	5’-CGTGGCAATGATCTCAACAC-3’	
Mouse CCL20	Sense	5’-GGTACTGCTGGCTCACCTCT-3’	57°C
	Antisense	5’-CACCCAGTTCTGCTTTGGAT-3’	
Mouse GAPDH	Sense	5’- ATCACTGCCACCCAGAAGAC-3’	57°C
	Antisense	5’- ATCACTGCCACCCAGAAGAC-3’	

Table S3. Antibodies

Antibodies	Company	Art . No .	Application
Mouse anti-human IFI16 antibody	Abcam	Ab55328	Western bloting (1:1000), Immunohistochemistry(1:200) Immunofluorescence (1:200)
Rabbit anti-mouse p204 antibody	Thermo Scientific	PA5-23494	Western bloting (1:1000), Immunohistochemistry(1:200)
Goat anti-human/mouse p65 antibody	Abcam	#6956	Western bloting (1:1000), Immunofluorescence (1:200)
Rabbit anti-human/mouse phosphor-p65 antibody	Cell Signaling Technology	#3033	Western bloting (1:1000), Immunofluorescence (1:200)
Rabbit anti-mouse CXCL10 antibody	Abcam	Ab9938	Immunohistochemistry(1:200)
Rabbit anti-mouse CCL20 antibody	Abcam	Ab9829	Immunohistochemistry(1:200)
Mouse anti-human STAT3 antibody	Cell Signaling Technology	#9139	Western bloting (1:1000)
Rabbit anti-human phosphor-STAT3 antibody	Cell Signaling Technology	#9145	Western bloting (1:1000)
Rabbit anti-human Erk1/2 antibody	Cell Signaling Technology	#4695	Western bloting (1:1000)

Rabbit anti-human phosphor-Erk1/2 antibody	Cell Signaling Technology	#4370	Western bloting (1:1000)
Rabbit anti- human TBK1 antibody	Abcam	Ab40676	Western bloting (1:1000)
Rabbit anti- human/ mouse β-actin antibody antibody	CWBIO	CW0097	Western bloting (1:2000)

Supplementary Figures

Supplementary Fig. S1. Psoriasis-related cytokines induce IFI16 expression in keratinocytes in a manner of dose and time dependent manner. (A-D) mRNA levels of IFI16 in human primary keratinocytes treated with different doses of IFN-γ, TNF-α, IL17 or IL22. **(E-H)** Keratinocytes were treated with IL-17A (20 ng/ml), IL22 (20 ng/ml), IFN-γ (10 ng/ml) or TNF-α (50 ng/ml) for 24 h. mRNA levels of IFI16 in these keratinocytes at 0, 6, 12, 24, 36 and 48 h. Values represent mean ± SD. *P < 0.05, **P < 0.01, ***P < 0.001, vs. 0 ng/ml; $^{\#}$P < 0.05, $^{\#\#}$P < 0.01, $^{\#\#\#}$P < 0.001, vs. 0 h.

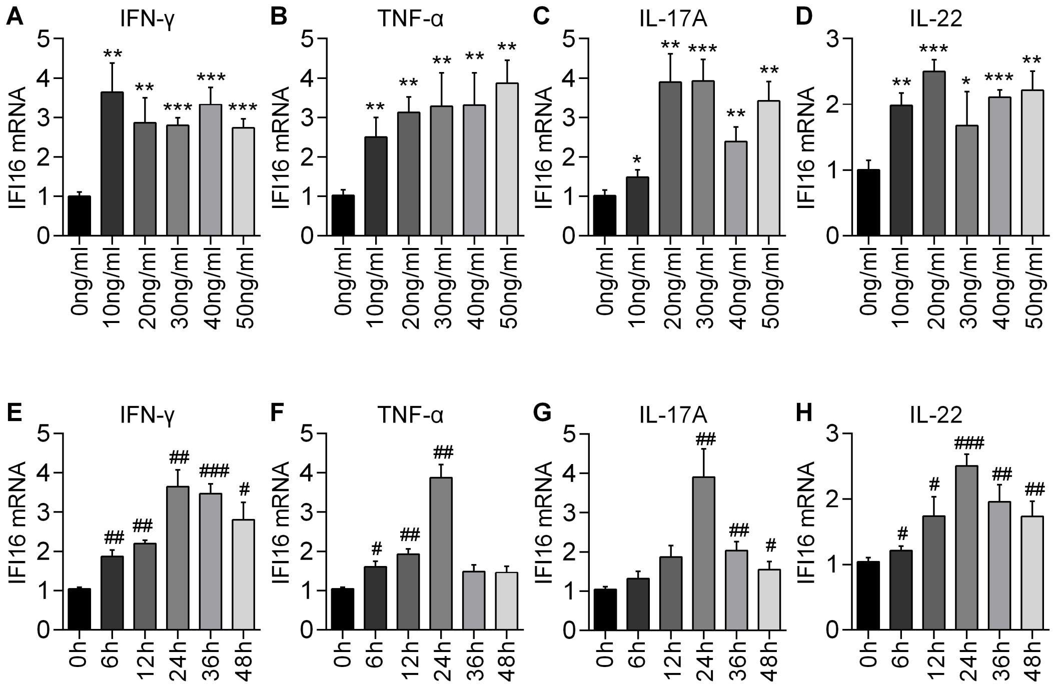

Supplementary Fig. S2. The efficiency of the inhibitors, siRNAs and plasmids. (A) The protein levels of Erk1/2 and the phosphorylation of Erk1/2 (p-Erk1/2) in human primary keratinocytes treated with the MAPK inhibitor for 1 h and then cocktail of cytokines for 24 h. **(B)** The protein levels of STAT3 and the phosphorylation of STAT3 (p-STAT3) in human primary keratinocytes treated with the STAT3 inhibitor for 1 h and then cocktail of cytokines for 24 h. **(C)** The protein levels of NF-κB p65 and the phosphorylation of NF-κB p65 (p-p65) in keratinocytes treated with the NF-κB inhibitor for 1 hand then cocktail of cytokines for 24 h. **(D)** The protein levels of IFI16 in keratinocytes treated with cytokine cocktail of and IFI16 siRNA or IFI16 plasmid for 24 h. **(E)** The protein levels of TBK1 in keratinocytes treated with TBK1 siRNA. β-actin was used as the internal standard.

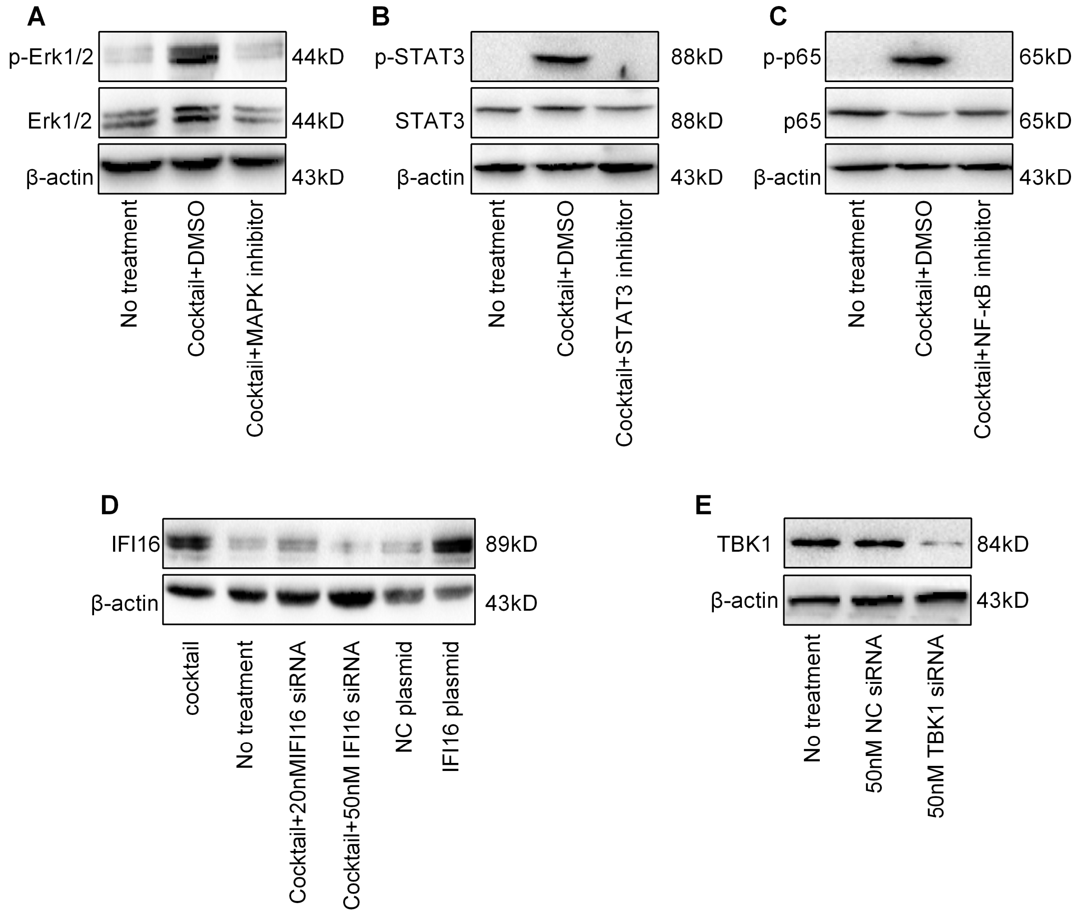
A
p-Erk1/2
44kD
Erk1/2
44kD
β-actin
43kD
No treatment
Cocktail+DMSO
Cocktail+MAPK inhibitor
B
p-STAT3
88kD
STAT3
88kD
β-actin
43kD
No treatment
Cocktail+DMSO
Cocktail+STAT3 inhibitor
C
p-p65
65kD
p65
65kD
β-actin
43kD
No treatment
Cocktail+DMSO
Cocktail+NF-κB inhibitor
D
IFI16
89kD
β-actin
43kD
cocktail
No treatment
Cocktail+20nMIFI16 siRNA
Cocktail+50nM IFI16 siRNA
NC plasmid
IFI16 plasmid
E
TBK1
84kD
β-actin
43kD
No treatment
50nM NC siRNA
50nM TBK1 siRNA

Supplementary Fig. S3. Overexpressed IFI16 does not promote keratinocyte proliferation. The proliferation of human primary keratinocytes with IFI16-knockin were detected using **(A)** CCK8, **(B)** MTT assay or **(C, D)** EdU staining. *P < 0.05, **P < 0.01, ***P < 0.001, vs. NC plasmid.

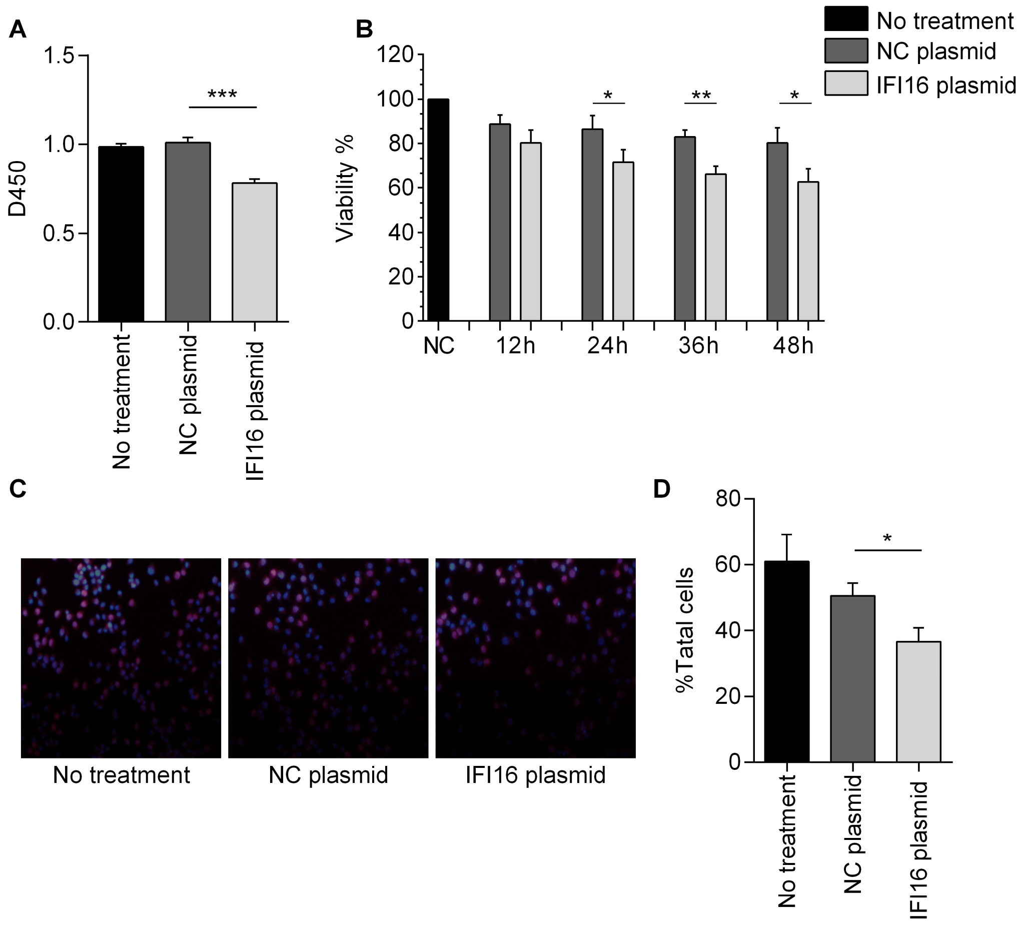

Supplementary Fig. S4. The effect of IFI16 on the expression of several cytokines and chemokines. (A-H) The mRNA expression levels of IFN-α1, IFN-α2, IFN-β, IL-6, IL-1β, IL-18, CXCL1 and CXCL16 in human primary keratinocytes treated with cytokine cocktail. Data presented are representative of three independently experiments. Values represent mean ± SD.

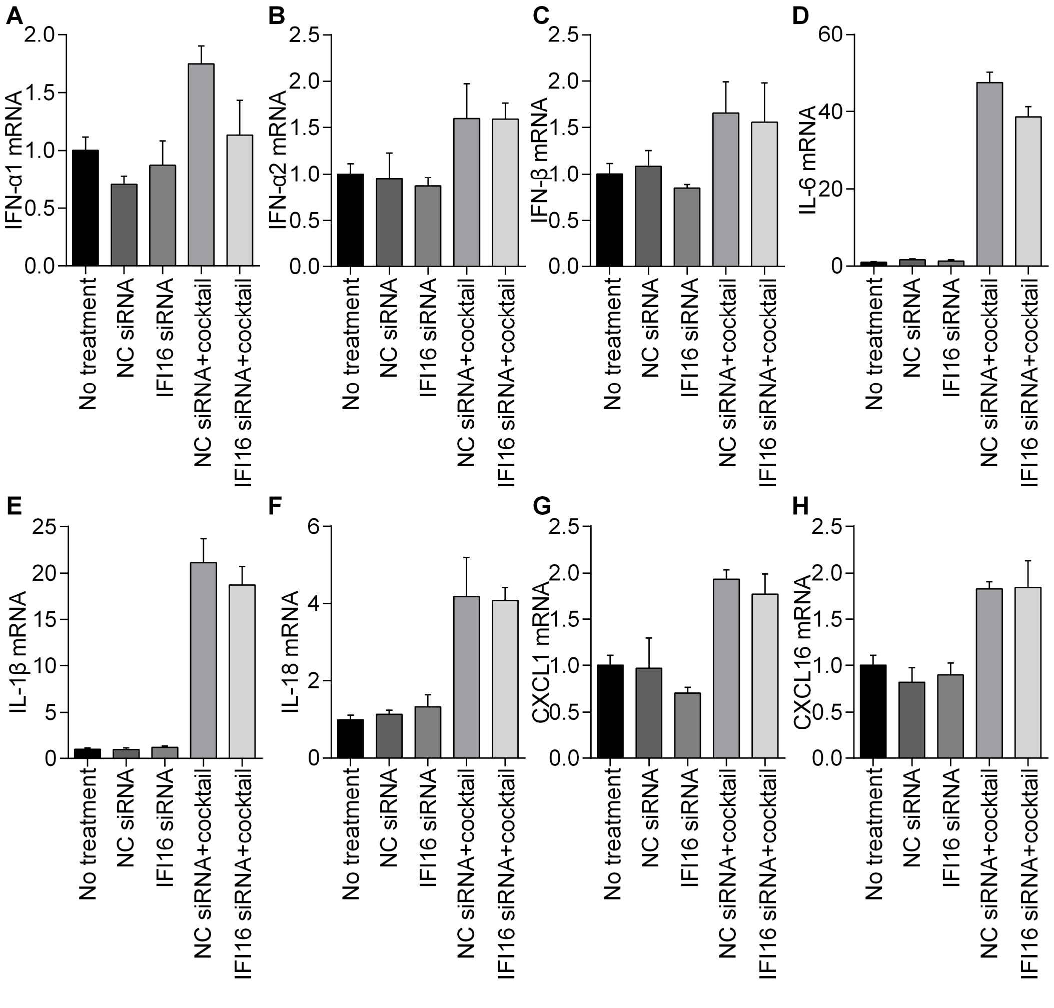

Supplementary Fig. S5. The p204 siRNA reduces the p204 expression in IMQ-induced psoriasis-like mice. The mice was treated with siRNA targeted p204 (2 nmol per ear) every 48 h and IMQ (10 ng per ear) every 24 h on the right ear, and NC siRNA on the left ear. **(A)** The protein levels of p204. **(B)** Immunohistochemistry staining of p204. Bar, 100 μm.

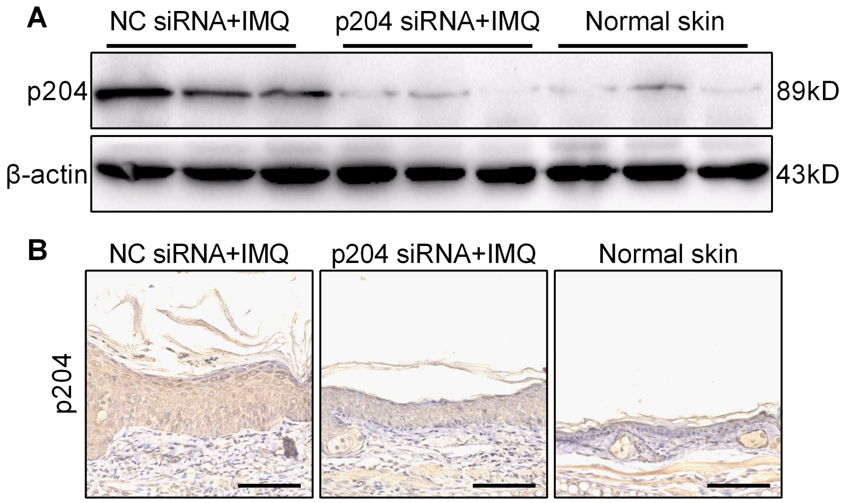

Supplement: Supplementary Information [file srep25381-s1.pdf]
